# Standardization of Minimally Invasive Tissue Sampling Specimen Collection and Pathology Training for the Child Health and Mortality Prevention Surveillance Network

**DOI:** 10.1093/cid/ciz565

**Published:** 2019-10-09

**Authors:** Natalia Rakislova, Fabiola Fernandes, Lucilia Lovane, Luisa Jamisse, Paola Castillo, Ariadna Sanz, Lorena Marimon, Susan Jesri, Melania Ferrando, Vima Delgado, Obdeningo Novela, Venceslau Muiuane, Mamudo R Ismail, Cesaltina Lorenzoni, Dianna M Blau, Quique Bassat, Clara Menéndez, Sherif R Zaki, Carla Carrilho, Jaume Ordi

**Affiliations:** 1 ISGlobal, Hospital Clinic, University of Barcelona, Barcelona, Spain; 2 Department of Pathology, Hospital Clinic, University of Barcelona, Barcelona, Spain; 3 Department of Pathology, Maputo Central Hospital, Maputo, Mozambique; 4 Faculty of Medicine, Eduardo Mondlane University, Maputo, Mozambique; 5 Center for Global Health, Centers for Disease Control and Prevention, Atlanta, Georgia, USA; 6 Centro de Investigação em Saúde de Manhiça, Maputo, Mozambique; 7 Catalan Institution for Research and Advanced Studies (ICREA), Barcelona, Spain; 8 Pediatric Infectious Diseases Unit, Pediatrics Department, Hospital Sant Joan de Déu, University of Barcelona, Barcelona, Spain; 9 Consorcio de Investigación Biomédica en Red de Epidemiología y Salud, Madrid, Spain; 10 Infectious Diseases Pathology Branch, Division of High-Consequence Pathogens and Pathology, National Center for Emerging and Zoonotic Infectious Diseases, Centers for Disease Control and Prevention, Atlanta, Georgia, USA

**Keywords:** minimally invasive autopsy, training, standardization

## Abstract

**Background:**

Minimally invasive tissue sampling (MITS) is a simplified postmortem examination technique that has shown to be an adequate approach for cause of death investigation in low-resource settings. It requires relatively low level of infrastructures and can be performed by health professionals with no background in pathology. A training program has been developed for the Child Health and Mortality Prevention Surveillance (CHAMPS) network to guarantee standardization of specimen collection techniques, procedures, and laboratory methods.

**Methods:**

The training program has included assessment of the site capacities and training on a standardized protocol of MITS sampling and histological processing. The project has also introduced a program of training for trainers for the personnel from Mozambique. To guarantee the adequacy of the procedure in each site, a trainer accompanied the local teams when the activities started. Training outcomes were assessed by evaluating the quality of the samples obtained and the quality of the slides produced locally.

**Results:**

Between June 2016 and October 2018, the laboratories of 7 sites (Bangladesh, Ethiopia, Kenya, Mali, Mozambique, Sierra Leone, and South Africa) have been evaluated and upgraded. Training has been delivered to 63 staff members from all sites. More than 600 MITS procedures have been performed. The quantity of tissue obtained in the MITS by the local teams was sufficient or abundant in 73%, and 87% of the slides were considered as technically acceptable or excellent.

**Conclusions:**

Satisfactory standardization of MITS and histology procedures has been achieved across all CHAMPS sites through organized capacity-building plans.

We describe and evaluate the training program and the methodology used to standardize the specimen collection techniques, procedures, and laboratory methods for the minimally invasive tissue sampling procedure across all CHAMPS network sites.

In 2013, the Barcelona Institute for Global Health (ISGlobal) started the validation study of the minimally invasive tissue sampling (MITS) against the complete autopsy (Cause of Death Determination Using Minimally Invasive Autopsy [CaDMIA] study), which was extended until the end of 2018 under the name CaDMIA-plus. The project was funded by the Bill & Melinda Gates Foundation and aimed at developing and validating a postmortem MITS procedure, also known as minimally invasive autopsy. The tool was developed as a proxy of the complete autopsy, the gold-standard method for cause of death investigation, and was specifically designed for low-resource sites [1–4]. The procedure consists of sampling of fluids and key organs using biopsy needles followed by histopathological and microbiological analysis of the obtained samples and can be rapidly performed by trained technicians close to the place where death occurs [1, 2]. The CaDMIA and CaDMIA-plus studies have validated the MITS tool against the complete autopsy for cause of death assignment in all age groups, including stillbirths, neonatal [5], pediatric [6], and adult deaths, including maternal deaths [7, 8], showing, even when used blindly to clinical data, a moderate to substantial concordance with the gold standard. The tool has been shown to be particularly useful for deaths due to infections and malignant tumors [5–7, 9]. A second major advantage of this approach is that it has been shown to be more acceptable than the complete autopsy by the relatives of the deceased individuals and by health professionals, in a variety of geographic, cultural, and religious backgrounds [10, 11]. These encouraging results have fueled the interest of funders and research groups toward the use of the MITS for cause of death investigations in sites where complete autopsies are unfeasible and/or unacceptable.

In 2015, the Child Health and Mortality Prevention Surveillance (CHAMPS) network was established as a long-term initiative funded by the Bill & Melinda Gates Foundation, with an objective of tracking the most preventable causes of mortality among children <5 years of age, utilizing the minimally invasive autopsy approach (newly rebranded as MITS) as a tool of choice for cause of death surveillance with the final aim of reducing under-5 mortality. Standardization of the laboratory methods at all CHAMPS sites was a priority since the conception of the project, as it was considered crucial to avoid differences in diagnoses/results between sites due to methodological differences. Additionally, a major objective of the project, centered in low- and middleincome countries (LMICs) with high under-5 mortality and limited resources, has been developing capacity in laboratory medicine, an essential component not only for achieving sustainability of the project, but also for enhancing the diagnostic capacities for the routine clinical activity [12, 13].

The objectives of such a standardization program were (1) to improve infrastructure, basic equipment, and supply chain management, and to assure laboratory supplies and equipment maintenance; and (2) to improve general skills of the local personnel to guarantee an adequate quality of the laboratory analyses. Specifically, we wished to ensure that the study staff (1) would be consistent in their assessment, recognition, and interpretation of external findings including anthropometric measurements; (2) adhere strictly to the standard operating procedures (SOPs) of the MITS; and (3) use standardized methods for handling and processing the samples. This paper describes the CHAMPS standardization program for the pathological methods, specific for stillbirths and child deaths, the training designed for site staff, and results on the performance of staff after the training.

## METHODS

### Standardized Operating Procedure for the MITS and MITS Kits

As part of the CaDMIA project, an SOP for the MITS specimen collection was developed at ISGlobal/Hospital Clinic/University of Barcelona, and later tested and refined at the Maputo Central Hospital (MCH) and the Centro de Investigaçao em Saúde de Manhiça (CISM), in Mozambique. The protocol has been the basis, with modifications, of the SOPs currently used in most of the proposals that have included MITS in their research, including the CHAMPS network project.

The MITS procedure, adapted for pediatric and neonatal/stillbirth age groups, can be performed by a single technician, helped by 1 or 2 assistants. No previous background in pathology is required of the personnel responsible of performing the procedure. In brief, the process starts with routine external body measurements (body length, mid-upper arm, head circumference, and length of foot and leg) and a detailed external inspection looking for potential congenital anomalies, evidence of trauma, tumors, skin lesions, or color changes, including maceration and umbilical cord abnormalities, in stillbirths. A detailed palpation of the abdomen and superficial lymph node areas is performed. Additionally, photographs of the body (front, back, side, face, and nails) are taken, including any distinctive external lesion. These photographs allow an eventual, retrospective evaluation of possible malformations or skin lesions by experts. Then, the surface of the body is carefully cleaned and disinfected, with emphasis on the areas where the puncture will be performed, to avoid contamination by skin microorganisms.

**Figure 1.**
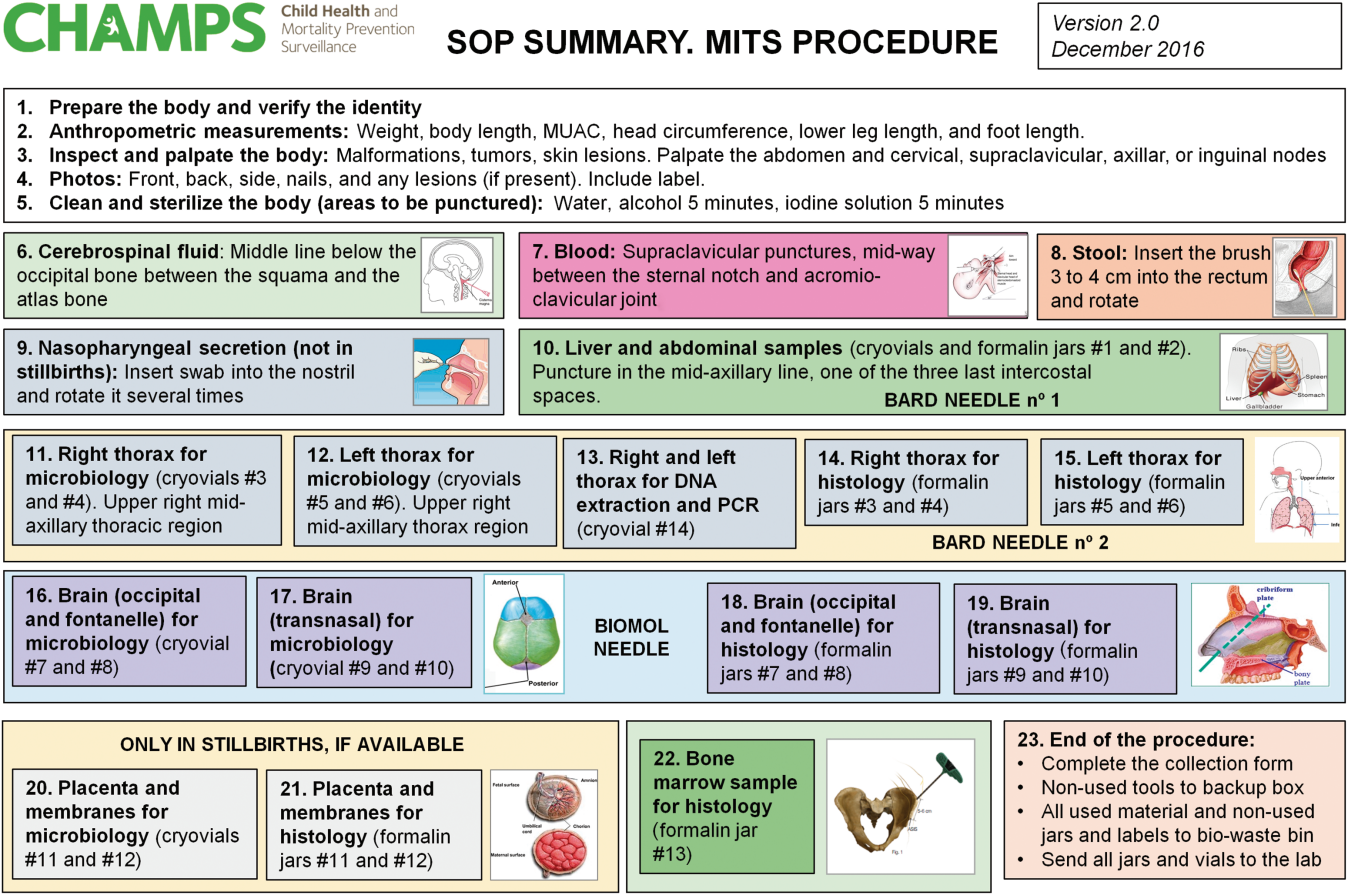
Summary of the standard operating procedure, including all the main tasks to be done during the minimally invasive tissue sampling. Abbreviations: MITS, minimally invasive tissue sampling; MUAC, mid-upper arm circumference; PCR, polymerase chain reaction; SOP, standard operating procedure.

**Figure 2.**
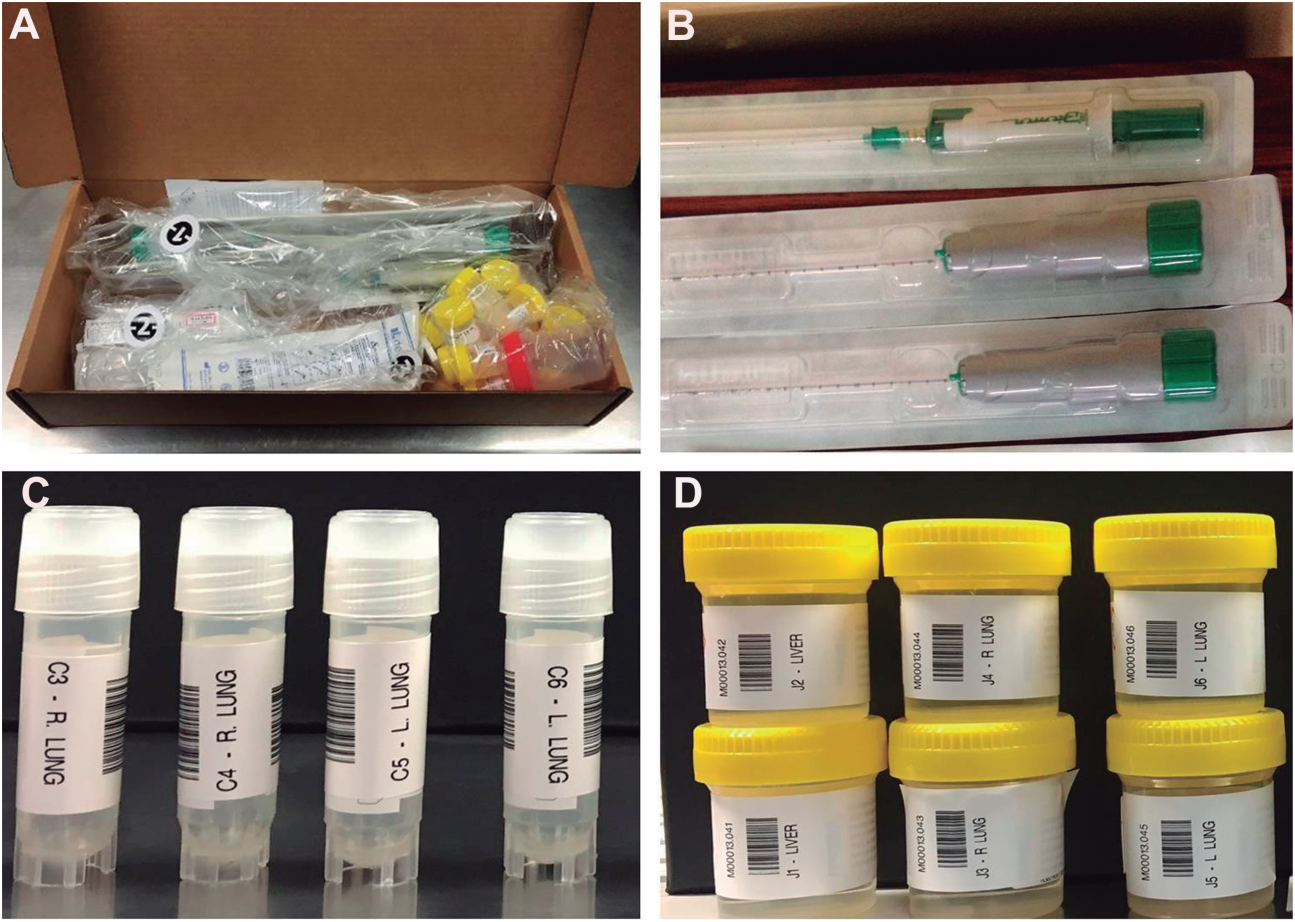
*A*, Minimally invasive tissue sampling (MITS) kit box including all the needles, tools, and containers required for MITS. *B*, Automatic needles used for the liver, lung, and central nervous system sampling. *C*, Prelabeled cryovials used for the tissue samples used for the microbiological analyses. *D*, Prelabeled containers with formalin used for the tissue samples used for histology.

The sampling starts with the collection of blood and cerebrospinal fluid (CSF), using pediatric needles, aiming to collect 10–20 mL of each fluid. CSF is obtained by occipital puncture, reaching the cisterna magna. Blood is collected by puncture of the subclavian vein through a supraclavicular approach. Additionally, a sample of stool is obtained using a rectal swab. Similarly, a sample of nasopharyngeal/oropharyngeal secretions is obtained using a swab. After the body fluids have been collected, samples from the liver, right and left chest targeting both lungs and heart, and the central nervous system (CNS) using 16G biopsy needles are obtained. The technicians are requested to obtain 6 samples from each organ, using the same entry point. The sampling should target different areas within the organ, by directing the needle to different regions, to obtain an adequate representation of the whole organ. In the lungs, the upper, middle, and lower fields are biopsied. For the CNS, 2 approaches are used: an occipital puncture, reaching the cranial cavity through space between the occipital bone and the first vertebra, and a transnasal approach, reaching the brain through the lamina cribrosa. In stillbirths, neonates, and young children, the anterior and posterior fontanelles are also used for the CNS sampling. In children >1 month of age, a bone marrow sample from the anterior iliac crest is obtained using a 12G bone trephine. In stillborn babies, the placenta is grossly examined, and samples of the placental tissue, the amniotic membranes, and the umbilical cord are collected for microbiology and pathology. Double sampling for histology is required in the protocol and 2 containers are obtained from each organ: one of them processed locally, and the second shipped to the central laboratory at the Centers for Disease Control and Prevention in Atlanta. The technicians are asked to complete a standardized form with the demographic data of the case, as well as the main characteristics of the sample collection process. A complete SOP, as well as a job aid with a summary of the SOP, is provided to the local team before the study initiation (Figure 1). All of the tools, containers, and forms included in each MITS kit box are prelabeled at ISGlobal (Barcelona) and sent to the sites ready-to-use, to guarantee adequate identification and tracking of the samples (Figure 2).

### Standardized Histological Processing and Evaluation of the Samples

The samples obtained for microbiological analysis are frozen at −80°C as soon as possible after the end of the procedure. Tissue specimens for histological analysis are fixed in 10% neutral buffered formalin for 4 hours to prevent antigen and nucleic acid degradation. After this short fixation, the samples are transferred into the cassettes and immersed in 70% ethanol until histological processing. Prelabeled histological cassettes and slides are included in each MITS box. All histological specimens are routinely embedded in paraffin. Four-micron sections are stained with hematoxylin and eosin (H&E) following standard procedures. Specimens are carefully handled to avoid loss of tissue during gross processing and sectioning. All samples taken during the MITS procedure are evaluated using light microscopy. All the H&E slides are initially evaluated by a trained local pathologist, who carefully counts the specimens present in each slide and records all the organs identified in the pathology evaluation, recording this information in a form specifically designed for the histological findings. After the initial histological evaluation, all the slides locally produced are scanned in a Hamamatsu NanoZoomer-SQ digital whole slide imaging scanner C13140-01 (Hamamatsu photonics, Tsukuba City, Japan). The scanned images are uploaded into a central server located in Atlanta.

### Assessment Visit and Site Preparation

**Figure 3.**
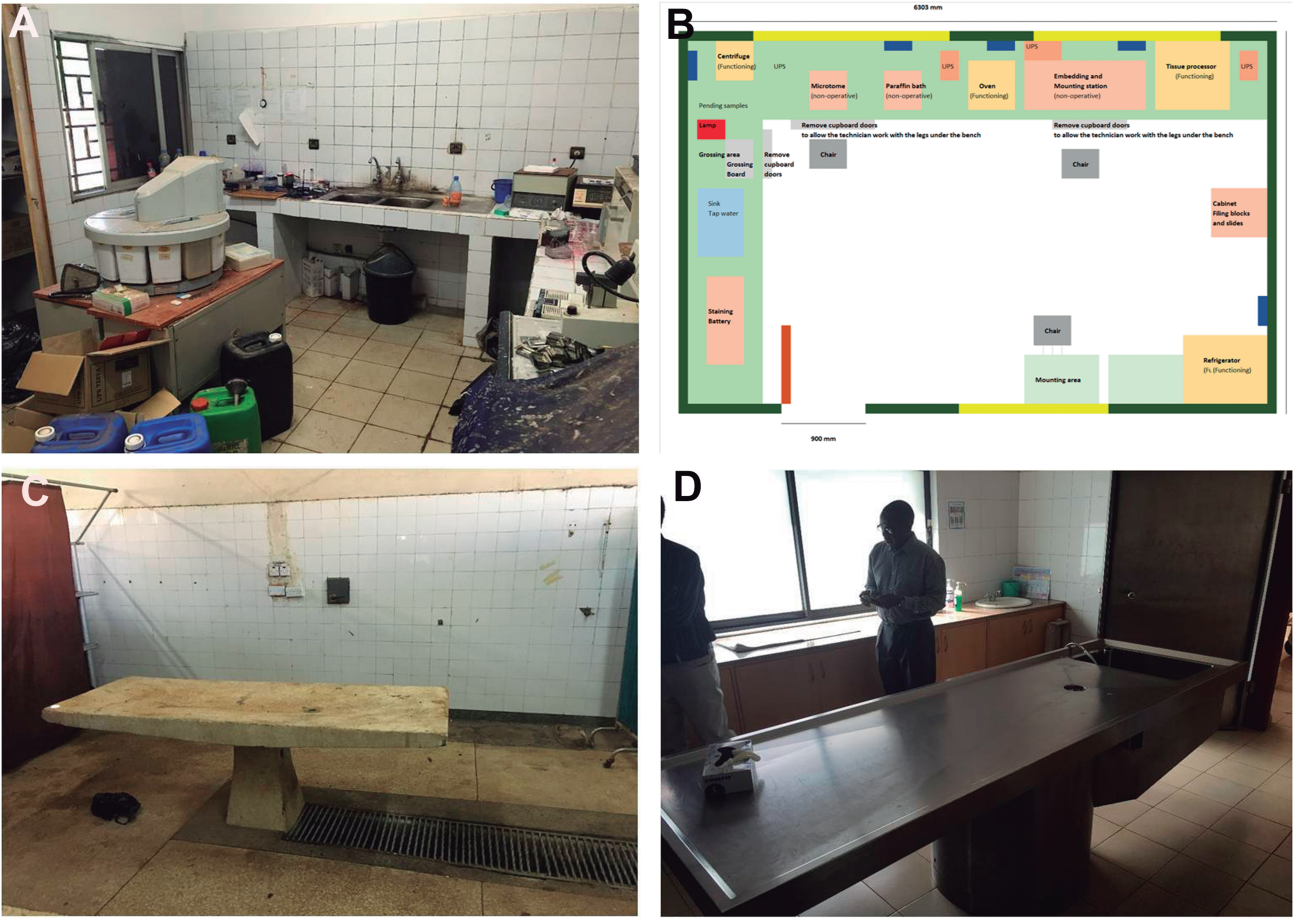
Assessment and upgrading of the pathology laboratories and autopsy rooms in the Child Health and Mortality Prevention Surveillance project. *A*, Pathology laboratory of one of the sites as it was found during the assessment visit. *B*, Upgrading plan designed for the pathology laboratory includes a floor map indicating the location of the new equipment. *C*, Autopsy room of one of the sites as it was found during the assessment visit. *D*, New autopsy room after the upgrade. Abbreviation: UPS, uninterruptible power supply.

Once the host country institution has signed the agreement to participate in the project and 4–5 months before the MITS specimen collection starts, an assessment of the site is done by the senior pathologist responsible for the training program. The visit is focused on the mortuary, the autopsy room, and the pathology laboratory, and its main objective is to assess the capacities of the site to implement MITS and to identify the needs for upgrading the pathology facilities, to reach the minimal quality requirements to implement the procedure. It also helps to identify the personnel to be involved in the MITS procedure and the histological processing and to determine the specific training needs. A report on the laboratory situation regarding capacities and needs, and an upgrading work plan are delivered to the local principal investigators and to the CHAMPS central office. The upgrading may include refurbishment of the facilities, acquisition of new equipment, and analysis and procurement of reagents needed, depending on the needs of each site (Figure 3). If required, a series of teleconferences are scheduled to follow up on the upgrading plan.

### Training Program on MITS Method and MITS Histology Processing

The main objective of the training plan is to guarantee that the MITS procedures are properly performed and all samples are correctly handled. In addition, the training helps to improve or acquire specific abilities of the medical and technical staff. The main training locations are Barcelona, Spain (ISGlobal/Hospital Clinic/University of Barcelona) and Maputo, Mozambique (MCH/University Eduardo Mondlane). Personnel from the Department of Pathology in the MCH were trained to conduct CHAMPS network pathology training during a preparatory phase in 2015. The training activities were tailored for each CHAMPS network surveillance site, both in terms of contents and duration, depending on the needs and capacities of each institution or laboratory.

The course on MITS method aims to train the local team on how to perform MITS, how to use the MITS kits and complete the MITS specimen collection forms, and how to guarantee the standardization and quality in the handling and processing of the specimens obtained by the MITS. The course is addressed to pathologists, other physicians, nurses, or other health professionals responsible for performing the MITS and is limited to 4 participants, to guarantee the practical component and personalized supervision. These courses, of 1-week duration, are conducted at the MCH.

The course on MITS histology technique aims to train the local team on the histological processing of the samples obtained at MITS and is based on practical sessions, focused on histological sectioning and staining of the samples obtained at MITS, with the objective of obtaining histological sections of sufficient quality. The instruction also includes basic training on how to assist the MITS specialist during the MITS procedure. The course is addressed to histology technicians and is limited to 2 participants per site. The courses, of 2-week duration, are conducted at ISGlobal/Hospital Clinic/University of Barcelona.

Training courses comprise lectures and practical sessions with real cases. The initial training lectures cover the background of the CHAMPS study, the performance and limitations of the MITS, the tools and containers used in the procedure, the rationale for standardization, a detailed description of the procedure, the general principles of the external examination and measurements, the examination of the placenta in stillborn babies and neonates, the basis of the processing of the samples, an initiation to digital pathology, and the use of scanners and general rules of biosafety. The courses for histology technicians additionally address basic aspects of fixation, processing, staining, and histochemical and immunohistochemical techniques. Training materials were prepared during the CaDMIA study and photographs taken by the training team. All training materials are in English.

### Evaluation of the Capacities of the Participants

At the end of each course on the MITS method, the attendants were requested to perform a MITS under supervision and the quality of the samples was evaluated by the trainer. Once the study started in each site, the quantity of the tissue collected from the MITS cases included in the study was evaluated by 1 of the ISGlobal trainers using the scanned images in 100 randomly selected slides produced at 4 of the local laboratories (Bangladesh, Ethiopia, Mali, and Mozambique [25 from each site]). The quantity of the collected samples was graded as (1) none (no targeted tissue identified); (2) scant (small specimens measuring in total <10 mm in length); (3) sufficient (specimens measuring in total 10–30 mm in length); and (4) abundant (specimens measuring in total >30 mm in length).

At the end of each course on MITS histology technique, the participants were requested to process 10 MITS samples independently, involving sectioning in a manual microtome and staining the slides with H&E. The quality of the slides was evaluated by the trainer. Once the recruitment of the cases starts in each site, the technical quality of the slides processed, sectioned, and stained locally was evaluated by 1 of the ISGlobal trainers, using the 100 randomly selected slides produced at the local laboratories used for the evaluation of the quantity of tissue. The quality of the histological slides was graded as (1) not evaluable; (2) poor (ie, evaluable, but showing marked artifacts hampering the evaluation; (3) acceptable (presence of artifacts that do not impair the histological evaluation); and (4) excellent (slides of good quality with no artifacts). The evaluation included fixation (eg, shrinkage, formalin pigment), sectioning (eg, thick sections, folds, wrinkles), and staining and mounting artifacts (eg, stain particles, bubbles, dust).

### Evaluation of the Training Program

All of the participants were invited to provide feedback on the training through a survey submitted to all of them immediately after the course. The questionnaire included questions related to (1) course contents (definition of the objectives of the training, relevance for the attendant, teaching level of the course; (2) faculty (preparation for the sessions, organization of the lectures, organization of the laboratory workshops, level of knowledge, teaching methods, encouragement of participation); and (3) general organization (facilities, convenience of the dates and times, general level of satisfaction with the course). The level of satisfaction was graded as 1 = very unsatisfied; 2 = unsatisfied; 3 = neither satisfied nor unsatisfied; 4 = satisfied; 5 = very satisfied.

### Initiation and Monitoring Visits

An initiation visit was scheduled when the MITS activities started at each site. This activity, typically of 3 weeks’ duration but tailored depending on the capacity of the site, was performed by a technician from ISGlobal, who visited the site when the activities started. The main objective of this visit was helping the research team on the kickoff of the MITS implementation, and supervising the adequacy of the sample collection, the histological processing, and the sample flow of the first MITS cases performed by the local team. If considered necessary in the initial assessment (limited capacity of the local laboratory or site team), the visit could be lengthened, include additional training on MITS technique or histology processing, and/or involve a pathologist. Finally, monitoring visits were scheduled, in the case they were considered necessary, with the basic objective of confirming that the MITS procedures and the histological technical process are conducted properly. Additionally, new local team members were trained if required.

**Ethical Considerations**

The study protocol and the design of the training courses was approved by the Clinical Research Ethics Committee of the Hospital Clinic of Barcelona, Spain (file number 2013/8677) and the National Bioethics Committee of Mozambique (reference number 342/CNBS/13). The training program was approved by CISM, the School of Medicine of Universidade Eduardo Mondlane, and MCH (all in Maputo, Mozambique) and ISGlobal (Barcelona, Spain) and was included in their institutional agreement (last amendment signed on 28 November 2017). MITS procedures were only conducted after verbal informed consent was provided by the relatives.

## RESULTS

Between June 2016 and October 2018, the pathology laboratories of 7 CHAMPS network sites (Bangladesh, Ethiopia, Kenya, Mali, Mozambique, Sierra Leone, and South Africa) have been evaluated and upgraded. Training courses on MITS method and MITS histology technique have been delivered to 14 and 10 staff members respectively, from the different sites. In addition, 39 staff members have been trained in MITS method during the initiation visits. The number and the background of the participants from each site to the MITS method and MITS histology technique courses, as well as the staff trained in the initiation visits, are shown in Table 1. All who attended the MITS technique courses successfully passed the final evaluation, being able to complete a MITS, by obtaining adequate samples and properly filling in the forms. Similarly, all those who attended the MITS histology technique courses successfully passed the final evaluation, being able to adequately process the MITS samples and produce slides of good quality.

**Table 1.** Background of Participants Attending Trainings on the Minimally Invasive Tissue Sampling Method and Histology Technique Conducted During the Preparatory Phase of Child Health and Mortality Prevention Surveillance

Site	Participants in MITS Method/MITS Histology Courses	Additional Staff Trained in Initiation Visits	Pathologists	Other Physicians	Other Health Workers	Other Professionals	Laboratory Technicians
Bangladesh	2/2	5	0	2	5	2	2
Ethiopia	2/2	2	0	2	0	0	2
Kenya	2/2	3	2	2	0	1	2
Mali	2/2	24	2	2	24	0	0
Mozambique	2/0	5	0	2	3	0	2
South Africa	2/0	0	0	2	0	0	0
Sierra Leone	2/2	0	2	0	0	0	2
Total	14/10	39	6	12	32	3	10

Abbreviation: MITS, minimally invasive tissue sampling.

Initiation visits were of 3 weeks’ duration in Bangladesh, Ethiopia, Kenya, and Mozambique, and 4 weeks in Sierra Leone and Mali. In Mali, 14 additional physicians, nurses, and technicians were trained during the initiation visit. An initiation visit was not considered necessary in South Africa because of the adequate technical capacity of the participating institution.

**Figure 4.**
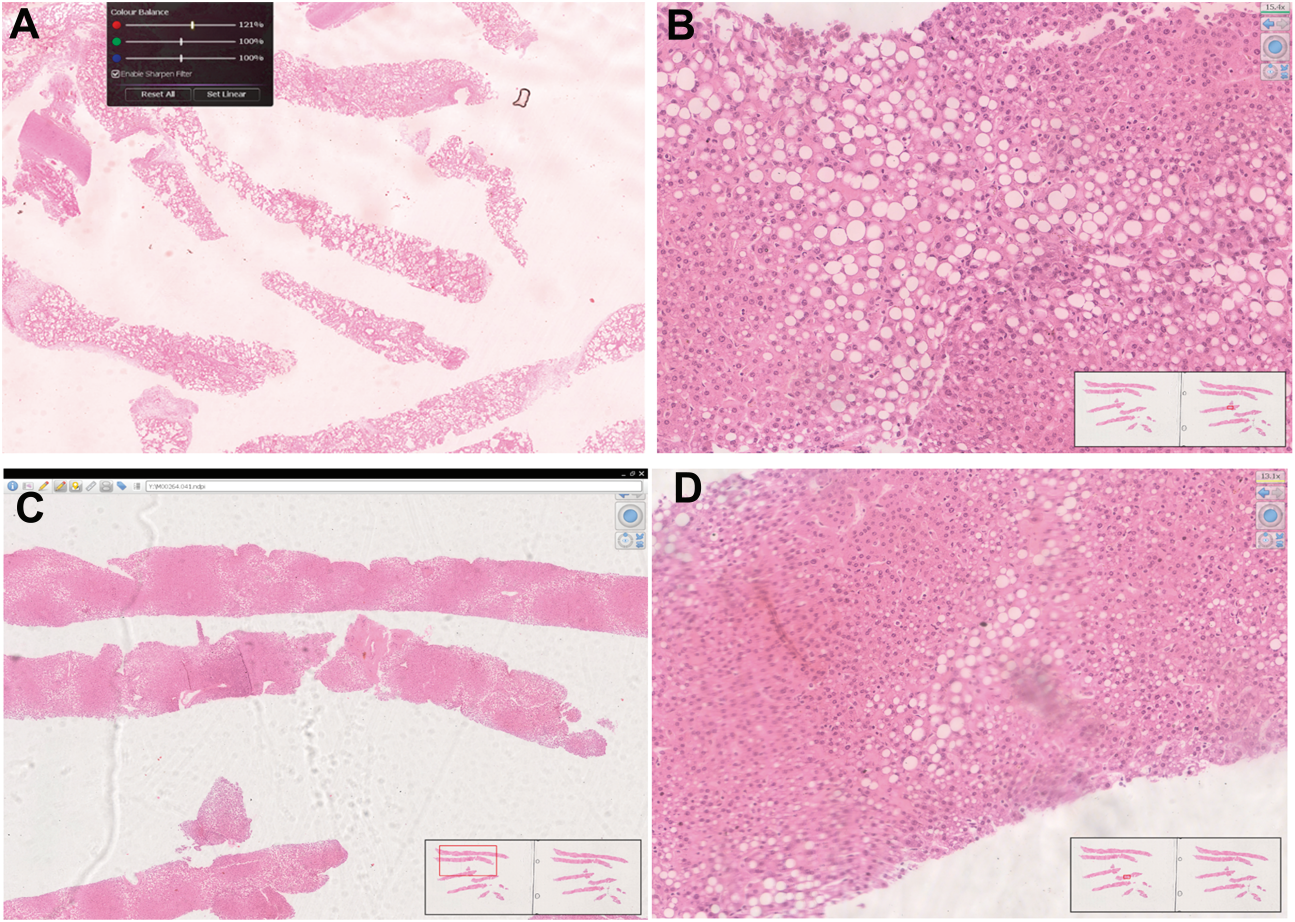
Representative photos of the scanned histological slides from different study sites. *A* and *B*, Excellent quality of the histopathological slides. *C* and *D*, Poor technical quality of the slides (extensive blurry areas hampering the evaluation).

In October 2018, >400 MITS had been performed. We evaluated the quantity of the collected samples for histology and the technical quality of the histological slides for 4 sites (Bangladesh, Kenya, Mali, and Mozambique) through the whole slide images scanned locally with the Hamamatsu scanner and uploaded into the central server located in Atlanta. Results from the period November 2016–June 2018 are shown in Table 2. The Ethiopia and Sierra Leone sites had not started MITS specimen collection when this study was prepared and consequently have not been evaluated. South Africa was not included either in the evaluation, as the slide scanner had not been installed yet. Overall, 73% of the tissue samples obtained in the sites were sufficient or abundant in terms of quantity of targeted tissue and 87% of the slides were acceptable or excellent in terms of quality of the histological processing. Examples of the quality of the samples and of the histological processing using remote evaluation of the scanned slides are shown in Figure 4.

**Table 2.** Quantity of the Collected Samples in the Minimally Invasive Tissue Sampling Procedures and Quality of the Produced Histological Slides in 4 of the Child Health and Mortality Prevention Surveillance Network Sites

Site	Quantity of the Samples				Quality of the Slides			
	No Sample	Scant	Sufficient	Abundant	Not Evaluable	Poor	Acceptable	Excellent
Bangladesh	1 (4)	6 (24)	15 (60)	3 (12)	0 (0)	1 (4)	17 (68)	7 (28)
Kenya	3 (12)	1 (4)	8 (32)	13 (52)	0 (0)	0 (0)	19 (76)	6 (24)
Mali	4 (16)	8 (32)	9 (36)	4 (16)	1 (4)	10 (40)	14 (56)	0 (0)
Mozambique	3 (12)	1 (4)	9 (36)	12 (48)	0 (0)	1 (4)	23 (92)	1 (4)
Total (n = 100)	11%	16%	41%	32%	1%	12%	73%	14%

Data are presented as No. (%) unless otherwise indicated. Twenty-five digitized slides were randomly chosen for each site (n = 100 slides).

The quantity of the samples is categorized as (1) no sample (no targeted tissue identified); (2) scant (small specimens measuring in total <10 mm in length); (3) sufficient (specimens measuring in total 10–30 mm in length); and (4) abundant (specimens measuring in total >30 mm in length).

The quality of the slides is categorized as (1) not evaluable (slides of poor quality, completely preventing evaluation); (2) poor (evaluable, but showing marked artifacts hampering the evaluation; (3) acceptable (presence of artifacts that do not impair the histological evaluation); and (4) excellent (slides of good quality with no artifacts).

Eleven of the 24 participants attending the training courses (46%) answered the satisfaction survey. Overall, 9 of 11 participants who answered the test were very satisfied with the training received and 2 of 11 were satisfied, regarding the course contents, professors/teaching, and organization of the courses. The mean score for the MITS method and for laboratory technique was 4.6 and 4.9, respectively.

## DISCUSSION

Staff training is an important component of any scientific project and is particularly necessary in projects focused in LMICs, where the number of trained personnel is limited and capacity building is more needed. However, most studies fail to document the contents of the training or to evaluate and report on its effectiveness. This training program is the one of the few large-scale initiatives focused on the specific postmortem sampling skills, including laboratory technique and diagnostic pathology training. Importantly, the program includes also a preparatory phase and accompanying training components. Previous initiatives that have included such a component, such as the training and quality control program of the Malaria in Pregnancy Consortium [14–17], conducted by our training team at ISGlobal, have not reported the results in detail. Other large-scale studies in low-resource settings are mainly focused on clinical standardization and training, with limited or no training specifically targeted to laboratory technicians and pathologists [18, 19].

Overall, the MITS specimen collection and pathology training program achieved a satisfactory level of standardization and staff training within and across the study sites, despite challenges including logistics, language differences, and intersite variation in the staff performing MITS procedures. Interestingly, 73% of the samples were sufficient or abundant in terms of quantity of targeted tissue and 87% of the slides were acceptable or excellent in terms of quality of the histological processing. However, some differences between sites were observed (Table 2). These differences are expected to diminish with time and as monitoring visits, specifically designed to minimize the variation across the sites, are conducted. The training effect was evident in our study and we observed laboratory capacity and diagnostic and technical skills improving rapidly. Some of the laboratories with limited prior experience in similar studies, such as the Kenyan and the Mozambican teams, became excellent performers. One of the main advantages of the MITS procedure is its simplicity, as it requires only a limited knowledge of anatomy and no specific skills in pathology or autopsy technique. Consequently, it can be performed by physicians, nurses, or other health professionals with no specific background in pathology. The main limitation of the tool is the potential of missing focal lesions, as the technique is designed to be performed in a blinded manner, and the possibility of insufficient tissue or fluid sampling, as in case of stillbirths. On the other hand, the current procedure is still excessively time-consuming and expensive for many local facilities, which hampers its routine implementation in resource-limited sites. In this regard, a simplified technique, involving an even more limited sampling than the conventional MITS approach, could contribute to the scale-up of MITS in rural high-mortality areas.

The CHAMPS site upgrading and capacity-building program has a number of strengths. Currently, both Maputo and Barcelona training sites have adequate facilities for training, including classrooms, autopsy room, and multiviewer teaching microscopes. Regular autopsies conducted in both hospitals offer excellent opportunities for practicing with real cases. Additionally, both teams are committed and have substantial experience in the technique and are able to solve most of the problems raised by the trainees. Finally, the implementation of a whole slide imaging scanner allows remote monitoring of the quality of the specimens and/or the histology technique. The implementation and utility of this equipment are discussed in detail by Martines et al elsewhere in this supplement.

Obviously, we faced challenges in this complex study involving diverse research sites. One of the main challenges of the training program has been the need of rescheduling the activities due to a variety of problems (eg, delays in improving laboratory structure, difficulties in obtaining the visas by the local personnel), which requires flexibility by the training teams. Language barriers, particularly with histology technicians, have also been occasionally encountered. Occasionally, the qualification of the participants was not adequate to the training objectives of the course (eg, pathologists attending the course on histology technique). Furthermore, training represents a significant workload for the Mozambican team, short of pathologists and technical staff, who frequently have to reschedule their routine clinical and teaching tasks, because of the training activities.

A future opportunity for the training program is to conduct part of the training through online courses, utilizing previously recorded video clips, webinars for online discussions, and quizzes of virtual slides and other course material. This approach would reduce the travel expenses and organizing efforts, limiting in-person training days only to acquire practical skills, mostly conduction of MITS and handling/processing of collected specimens.

Unfortunately, pathology services in LMICs are scarce and the existing facilities face serious daily difficulties including inadequate infrastructure, lack of quality standards, insufficient human resources, and inadequate training [12]. Scientific projects may not have the long-term desirable effect, if these unsolved difficulties persist in time, hampering the implementation of new techniques and projects. Thus, it is urgent to find solutions to all these barriers to ensure future implementation and successful long-term effect of our training program [13]. The CHAMPS project has addressed some of these limitations. The project has upgraded the local laboratories when needed, providing technical support for instrumentation and supply of reagents to guarantee not only the adequate technical processing of the project samples, but also helping to maintain the routine clinical activity of the health centers participating in the study. In addition, the project has supported stable internet connection, crucial to maintain the quality assurance and to provide continuous training of the pathologists involved in the study. The project has designed and implemented a training plan aiming not only at guaranteeing the proper performance of the MITS procedure, but also to help the medical and technical staff to improve their technical and diagnostic abilities, one of the main limitations in LMICs [12]. Finally, CHAMPS has provided standardized procedures and has established the necessary quality control of these implemented procedures in the project.

We hope that the methods, results, and challenges/lessons learned from the CHAMPS training program will be useful for other large-scale studies focusing on mortality in LMICs. As well as providing confidence in the CHAMPS methods, our experience indicates that multisite studies requiring good laboratory equipment and human resources can be undertaken at research sites with limited prior experience and equipment.

## Notes

***Financial support.*** This work was supported by the Bill & Melinda Gates Foundation [OPP1126780]. The CaDMIA research project (validation of the minimally invasive autopsy tool for cause of death investigation in developing countries) and the CadMIA-plus research and training project were funded by the Bill & Melinda Gates Foundation (global health grant numbers OPP1067522 to Q. B. and OPP1128001 to J. O.). ISGlobal is a member of the Centre de Recerca de Catalunya program, Generalitat de Catalunya.

***Supplement sponsorship. ***This supplement is sponsored by the Emory Global Health Institute and the Bill & Melinda Gates Foundation.

***Potential conflicts of interest.*** All authors report no potential conflicts. All authors have submitted the ICMJE Form for Disclosure of Potential Conflicts of Interest. Conflicts that the editors consider relevant to the content of the manuscript have been disclosed.
